# The pressure-enhanced superconducting phase of Sr$_x$–Bi$_2$Se$_3$ probed by hard point contact spectroscopy

**DOI:** 10.1038/s41598-021-83411-w

**Published:** 2021-02-18

**Authors:** Ritesh Kumar, Aastha Vasdev, Shekhar Das, Sandeep Howlader, Karn S. Jat, Prakriti Neha, Satyabrata Patnaik, Goutam Sheet

**Affiliations:** 1 Department of Physical Sciences, Indian Institute of Science Education and Research (IISER) Mohali, Sector 81, S. A. S. Nagar, Manauli, 140306 India; 2 School of Physical Sciences, Jawaharlal Nehru University, New Delhi, India

**Keywords:** Topological insulators, Phase transitions and critical phenomena, Superconducting properties and materials

## Abstract

The superconducting systems emerging from topological insulators upon metal ion intercalation or application of high pressure are ideal for investigation of possible topological superconductivity. In this context, Sr-intercalated Bi$_2$Se$_3$ is specially interesting because it displays pressure induced re-entrant superconductivity where the high pressure phase shows almost two times higher $T_c$ than the ambient superconducting phase ( $T_C\sim 2.9$ K). Interestingly, unlike the ambient phase, the pressure-induced superconducting phase shows strong indication of unconventional superconductivity. However, since the pressure-induced phase remains inaccessible to spectroscopic techniques, the detailed study of the phase remained an unattained goal. Here we show that the high-pressure phase can be realized under a mesoscopic point contact, where transport spectroscopy can be used to probe the spectroscopic properties of the pressure-induced phase. We find that the point contact junctions on the high-pressure phase show unusual response to magnetic field supporting the possibility of unconventional superconductivity.

## Introduction

In superconductors, due to particle-hole symmetry, the positive and negative energy eigenstates of the Bogoliubov–de Gennes Hamiltonian come in pairs^1,2^. In the superconducting ground state, the negative-energy eigenstates are fully occupied. Therefore, as in case of insulators, depending on the dimension and the symmetries of the system, various topological numbers (e.g., the Chern number) for the occupied states can be defined^3–6^. If non-zero topological numbers exist for a superconductor, that can be classified as a “topological” superconductor^7–10^. By this definition, when certain unconventional superconductors display nodes in the order parameter symmetry, the node themselves might have non-zero topological numbers thereby making the superconductors “weakly” topological. On the other hand, in strong topological superconductors, the non-zero topological numbers can exist along with a fully gapped bulk superconducting gap. Hence, characterizing the topological nature of strong topological superconductors is a challenging task. However, due to topological restrictions, the surface of such superconductors host gap-less modes which can be detected by surface sensitive spectroscopic techniques^11–15^. Potentially, point-contact Andreev reflection can be a powerful technique to probe transport through such topological surface states in a topological superconductor^13,16–18^. One popular route to possibly achieving topological superconductivity is doping charge carriers through metal intercalation in topological insulators like Bi$_2$Se$_3$^19–22^. ARPES experiments have confirmed that at the required doping level for superconductivity ($\sim 2 \times 10^{20}$ cm$^{-3}$) in charge doped Bi$_2$Se$_3$ systems, there is still significant separation in the momentum space between the topological surface states and the bulk states^22^. Hence, it is expected that when the bulk superconducting phase leads to proximity-induced superconductivity on the surface, due to the inherent topological nature of the surface states, the proximity induced phase should become a 2D topological superconductor^10,14,15,23^. Another potentially interesting way of inducing superconductivity in a topological insulator is through applying pressure^24–27^. A pressure-induced superconducting phase was indeed found in undoped Bi$_2$Se$_3$^27^. A more interesting pressure-induced superconducting phase was seen to appear in Sr-intercalated Bi$_2$Se$_3$ which shows ambient superconductivity below $T_c = 2.9$ K^28^. In this case, superconductivity was first seen to disappear with applying pressure and re-emerge at higher pressure^26^. The high-pressure re-entrant superconducting phase was found to be interesting owing to a significantly higher $T_c$ compared to the $T_c$ of the ambient superconducting phase of Sr–Bi$_2$Se$_3$. More importantly, the pressure-induced re-emerged phase showed strong signatures of unconventional superconductivity indicating a high possibility of the pressure-induced superconducting phase of Sr–Bi$_2$Se$_3$ being topological in nature. However, because technologically it is extremely challenging to perform spectroscopic investigation of the re-entrant phase, the exact nature of superconductivity in this phase remained poorly understood. In this paper, we discuss a unique way of realizing such a superconducting phase by applying uniaxial pressure under a point contact, where the superconducting phase can be investigated through mesoscopic transport spectroscopy.

## Results and discussion

**Figure 1:**
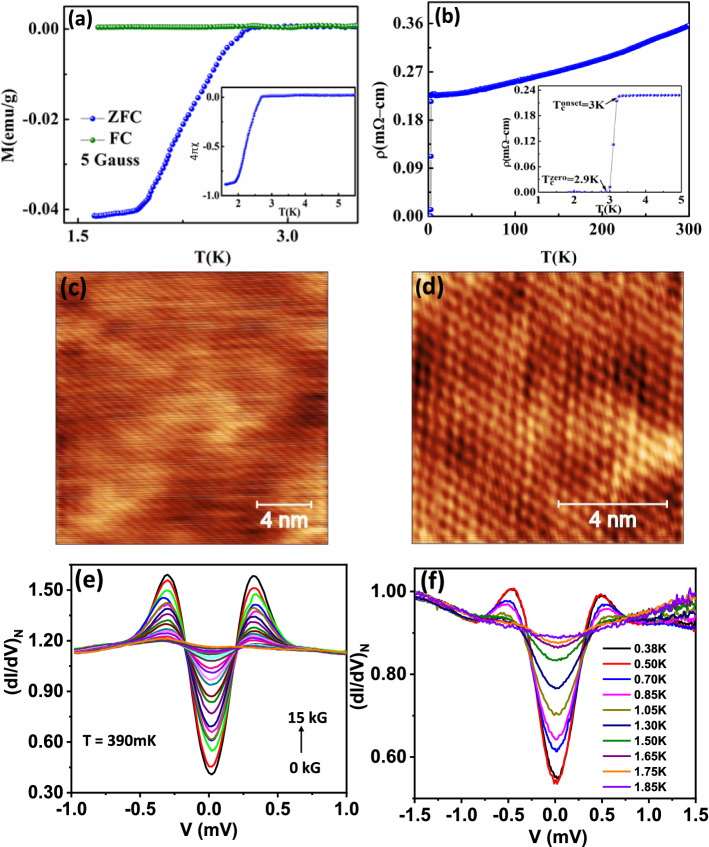
(**a**) Magnetization vs temperature (Zero field cooled (ZFC) and Field cooled (FC)). (**b**) Resistivity ($\rho$) as a function of temperature (*T*) of Sr$_{0.1}$Bi$_2$Se$_3$. (**c**) STM topography of the Sr$_{0.1}$Bi$_2$Se$_3$ cleaved surface (20 nm $\times$ 20 nm). (**d**) Atomic resolution image (10 nm $\times$ 10 nm) of the Sr$_{0.1}$Bi$_2$Se$_3$ surface. (**e**) Temperature dependence of the STS spectra upto 1.85 K. (**f**) Normalized STS spectra with varying magnetic fields upto 15 kG.

We have performed experiments on high quality single crystals of Sr$_{0.1}$Bi$_2$Se$_3$. The bulk magnetization (Fig. 1a) and transport measurements (Fig. 1b) revealed a critical temperature $T_c\sim 2.9$ K below which the system superconducts. Here the magnetic transition appears to be broad. We have also included the magnetization data in Fig. 1 in the “Supplemental material” with field applied in plane where a sharper transition is seen. It should be noted that unlike resistive transition (which is sharp with a width of 0.1 K; please see Ref.^19^) the magnetic transition reflects gradual flux penetration.

**Figure 2:**
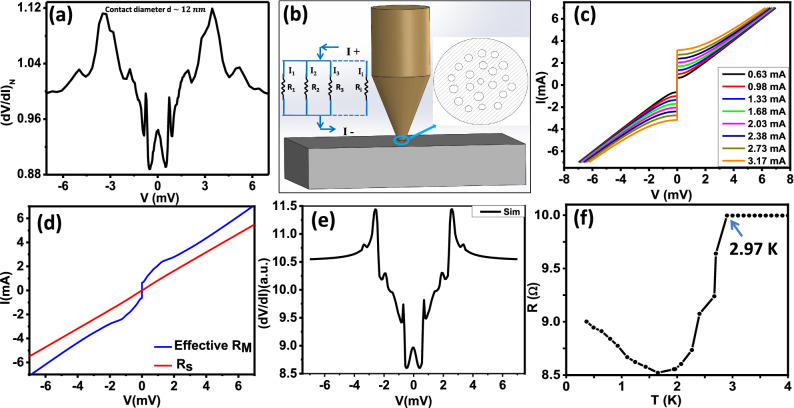
(**a**) Normalized ($(dV/dI)_N$) spectrum obtained in the intermediate regime of transport showing the characteristic signatures of critical current peaks and Andreev reflection (AR) dips. (**b**) Schematic of point contact setup. Inset shows the assembly of microconstriction of different diameter and the effective electrical circuit. (**c**) *I*–*V* characteristics corresponding to Maxwell's resistance for different values of $I_C$. (**d**) *I*–*V* characteristics corresponding to Sharvin's resistance calculated from the BTK model (red) and effective Maxwell's resistance (blue). (**e**) Calculated *dV*/*dI* vs *V* spectrum of the modelled electrical circuit. (**f**) Zero bias resistance (*R*) vs. *T* of the point contact in intermediate regime.

The quantitative analysis of such measurements is marred by demagnetization factor that depends on the geometry of the sample vis a vis the applied field direction. We have analyzed the pinning properties and provided the details in Fig. 2 in the “Supplemental material”. These materials generally have very small $H_{c1}$ (lower critical field). In particular, as the temperature is increased from the lowest temperature (ZFCW), once $H_{c1}$ is crossed, the rate of external field penetration depends on pinning (impurities, defects) strength. In this sense, a broader ZFC implies a purer crystal. This is also evident in our Field cooled data (Fig. 1a); negligible pinning and thus hardly any diamagnetic flux trapping. Furthermore, similar broad magnetic transitions are observed in single crystals of other (topological) superconducting materials as well^13,20^.

The high quality of the crystals was further confirmed by scanning tunnelling microscopy and spectroscopy. As shown in Fig. 1c,d, the atomic lattice is seen with very low defect density. Tunnelling spectroscopy revealed a fully formed superconducting gap that evolves systematically with increasing temperature and near 2 K the spectra become too broad for the gap to be clearly seen (Fig. 1f). The gap also evolves systematically with magnetic field before being almost completely suppressed at 15 kG (Fig. 1e).

**Figure 3:**
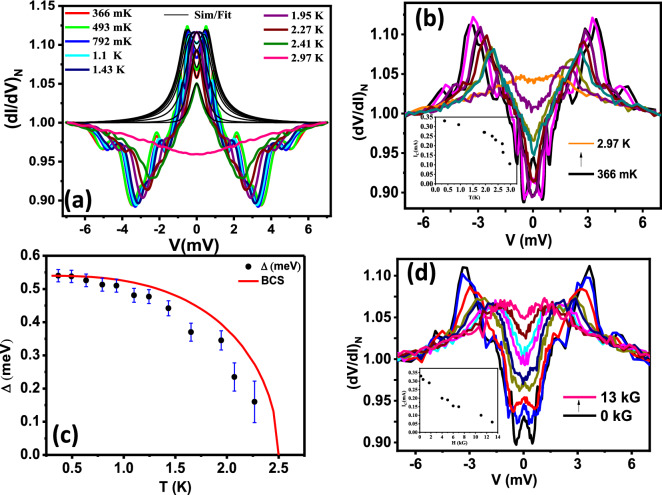
(**a**) Temperature dependence coloured dots) of normalized (dI/dV)_N vs V along with the best theoretical BTK fits (solid black lines) of the spectra shown in Fig. 2a. (**b**) Temperature dependence of the spectrum shown in Fig. 2a. The *inset* shows temperature dependence of the current corresponding to the peaks ($I_c$) extracted from the data. (**c**) $\Delta$ vs. *T* plot extracted from (**a**). The Red line shows the expected BCS line while the black dots show the experimental data (**d**) Magnetic field (*H*) dependence of the spectrum shown in Fig. 2a. The *inset* shows magnetic field dependence of the current corresponding to the peaks ($I_c$) extracted from the data.

These observations for the ambient superconducting phase of Sr–Bi$_2$Se$_3$ are consistent with the previous experiments^28^. We have fitted the STS data and the estimated superconducting energy gap is 0.31 meV (Fig. 5 in “Supplemental material”). Here our aim was to probe the pressure-induced re-entrant, enhanced superconducting phase of Sr–Bi$_2$Se$_3$. For that we used a home-built point contact spectroscopy set up working down to 400 mK^29^ in another $He^{3}$ cryostat. First a silver (Ag) tip was engaged gently on the surface of the crystal thus forming mesoscopic point contact junctions between Ag and Sr–Bi$_2$Se$_3$. The smallest area point contacts revealed dips in *dV*/*dI* vs. *V* spectra (Fig. 2a) showing signature of Andreev reflection. A visual inspection itself reveals that in addition to the features associated with Andreev reflection, multiple peaks in *dV*/*dI* also appeared at different values of the dc bias. Such features can appear when the point contact is slightly away from the pure ballistic regime, but in an intermediate regime. In order to make the origin of the multiple peak structures clear, we have modelled a point contact in intermediate regime where there are multiple micro-constrictions appear under the contact and electrical transport takes place through those constrictions. Figure 2b shows a schematic of the assembly of micro-constrictions that have been used for analysis. The effective electrical circuit for such a contact can be represented by a number of resistors connected in parallel configuration as shown in inset of Fig. 2b. Now, since the micro-constrictions are part of a superconducting point-contact, if the diameter of some of them is large, they will contribute a resistance which is approximately equal to the Maxwell’s resistance ($R_M$)^30^ as obtained in thermal limit of transport. The *I*–*V* characteristics corresponding to such “thermal-like” constrictions are dominated by the critical current ($I_c$). Each constriction will have a different value of $I_c$ because there is no reason why they should be all geometrically identical. In Fig. 2c, we show the representative critical current dominated *I*–*V* characteristics for a collection of micro-constrictions. Now, since some of the constrictions are also in the ballistic regime (as confirmed by the observation of Andreev reflection related features), the Andreev reflection dominated *I*–*V* characteristics (corresponding to the Sharvin’s resistance ($R_S$)^30^) will also be shown by the point contact under which such micro-constrictions have formed. Adding all these components in different proportions, we have calculated the resulting *I*–*V* characteristics and *dV*/*dI* vs. *V* spectrum which is shown in Fig. 2d,e respectively. The striking similarity of the calculated spectrum (Fig. 2e) with the experimentally obtained spectrum (Fig. 2a) shows that the multiple peaks in *dV*/*dI* indeed appeared from multiple critical currents under the point contact. In Fig. 2f, we show the temperature dependence of the point contact resistance which shows a transition at 2.9 K indicating that the low-pressure superconducting phase of Sr–Bi$_2$Se$_3$ has been probed by the point contact.

**Figure 4:**
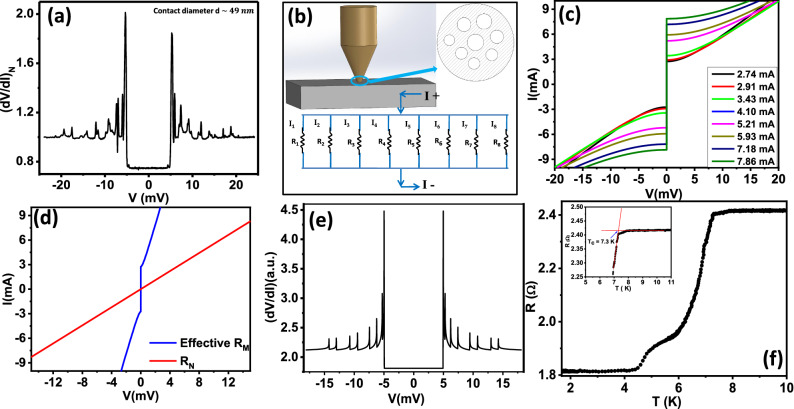
(**a**) Normalized differential resistance ($(dV/dI)_N$) spectrum in the thermal regime showing the multiple critical current peaks. (**b**) Schematic of point contact setup with microconstrictions of different diameter and modelled electrical circuit in thermal regime. (**c**) *I*–*V* characteristics of Maxwell's resistance for different values of $I_c$. (**d**) *I*–*V* characteristics corresponding to effective Maxwell's resistance ($R_M$) (blue) and normal point contact resistance ($R_N$)(red). (**e**) Calculated *dV*/*dI* vs *V* spectrum. (**f**) Resistance (*R*) of the point contact vs. *T* in the thermal regime. The *inset* shows the method of estimation of $T_c$.

Since this spectrum also contains clear signature of Andreev reflection, we attempted to fit the lower-bias part of the spectrum by the theory developed by Blonder–Tinkham–Klapwijk (BTK) ignoring the critical current driven effects. A typical fit is shown in Fig. 3a and the fitting parameters for all the spectra is shown in Table I in the “Supplemental material”. From such fitting, the superconducting energy gap ($\Delta$) is found to be 0.54 meV. Then, we increased the temperature and by analysing the temperature dependent spectra (Fig. 3b) in the similar way, we plotted $\Delta$ vs. *T* (Fig. 3c) which approximately followed the temperature dependence as predicted within BTK formalism (red line in Fig. 3c). Further, we varied the magnetic field and found that the spectral features related to superconductivity disappear at 13 kG as shown in Fig. 3d. The position of the dominant peak corresponding to critical current is plotted in the *inset* of Fig. 3d. As expected, the critical current systematically decreased with magnetic field.

**Figure 5:**
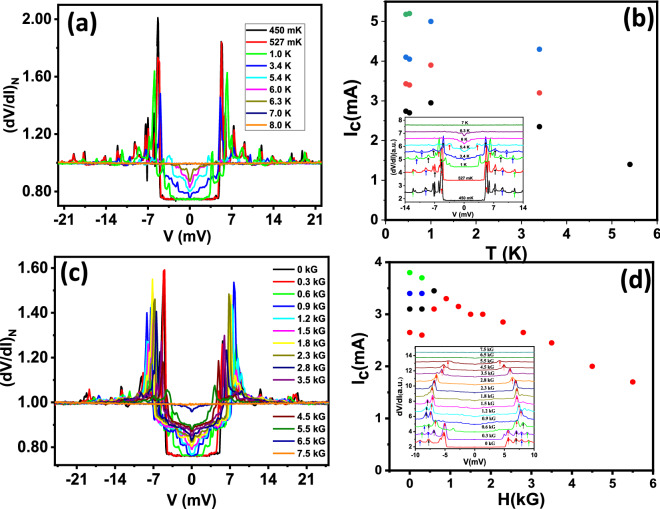
(**a**) Temperature dependence of the spectrum shown in Fig. 4a. (**b**) Temperature dependence of the critical current corresponding to the peaks structure ($I_c$) vs. *T* extracted from the data in (**a**). Inset shows the critical current peak position marking with coloured arrows. (**c**) Magnetic field dependence of the spectrum shown in Fig. 4a. (**d**) Magnetic field dependence of the critical current corresponding to the peaks structure ($I_c$) with *H* extracted from the data in (**c**). The *inset* shows the multiple critical current peaks (marked with coloured arrows).

**Figure 6:**
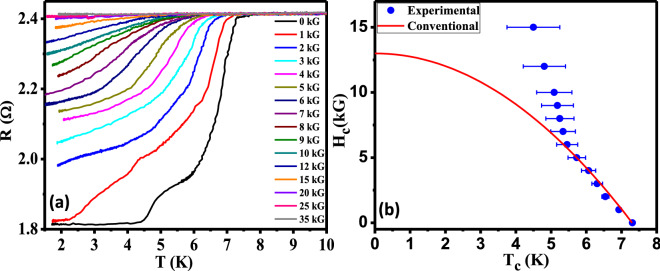
(**a**) Magnetic field dependence of the *R-T* curves. (**b**) *H-T* phase diagram. The red line shows the empirical plot for a conventional superconductor. The blue dots are the data points extracted from the curves in (**a**).

After that, we continued pressing the tip harder onto the crystal surface. During the process, we found signature of superconductivity at a temperature higher than the $T_c$ of pristine Sr–Bi$_2$Se$_3$. Upon applying large pressure under the point contact, superconductivity at a higher temperature is achieved, but now due to large force applied, the contact diameter also changed thereby forming a point contact in the thermal regime of transport. In this extreme thermal regime, features associated with Andreev reflection completely disappeared.As it is seen in Fig. 4a, multiple sharp peaks in *dV*/*dI* appeared indicating formation of multiple thermal point contacts under the contact region. However, the overall shape of the spectrum is completely different from the spectrum obtained in the intermediate regime. In order to demonstrate the origin of the flat low-bias part of the spectrum and the multiple sharp peaks, we modelled a thermal regime point contact where multiple constrictions cause non-linearity in the *I*–*V* characteristics due to their respective critical currents (as shown in Fig. 4c). Furthermore, since no spectral feature associated with Andreev reflection is seen, we simply added a linear *I*–*V* originating from the non-superconducting components of the point contact (as shown in Fig. 4d). This is not the ideal case scenario, but nevertheless is a good approximation. After adding all these components in different proportions, we calculated the *dV*/*dI* vs. *V* for the point contact. As shown in Fig. 4e, the spectral features as observed in the experimental point contact spectrum have been reproduced within our multiple critical-current based model (Fig. 4b).

A temperature dependent measurement of the point contact resistance revealed a critical temperature $T_c\sim 8$ K which corresponds to the known pressure-induced re-entrant superconducting phase of Sr–Bi$_2$Se$_3$^26^. As it is seen in the point contact *R*–*T* data, the transition is broad and at a relatively lower temperature (around 6 K), another transition like feature is seen. These could be attributed to multiple electrical contacts with different contact geometries formed. Each contact may experience different pressure due to the difference in their effective contact area. This is consistent with the idea that electrical transport in a point contact happens through multiple conducting constrictions. Comparing the measured $T_c$ with the published literature^26^, we estimate the approximate pressure experienced by the superconducting region under the point contact to be approximately 15 GPa.

In order to gain further understanding on the pressure-enhanced superconducting phase, we carried out detailed temperature and magnetic field dependent experiments. As seen in Fig. 5a, the point contact spectrum evolves monotonically with temperature. At the lowest temperature (450 mK), the critical-current dominated features (peaks in *dV*/*dI*) are extremely sharp (in Fig. 5b). With increasing temperature, all the peaks shift and they come closer indicating temperature dependent suppression of critical current for each micro-constriction formed under the point contact. Finally, at 8 K, all the features associated with superconductivity disappear. The critical current driven peaks also shift inward with increasing magnetic field (Fig. 5c). For all the micro-constrictions, the critical current is seen to decrease at a slow rate. For the constriction with highest critical current (red dots in Fig. 5d), the critical current shows slight increase at lower fields and then starts decreasing slowly. At a field of 6 kG, the critical current has become only half of the zero field value. The over-all superconductivity-related spectral features completely disappear at 7.5 kG. Therefore, it is seen that the magnetic field dependence of critical current for the high-pressure phase is significantly different from that in the low-pressure phase (*inset* of Fig. 3b). In fact, the low-field behaviour of the critical current seems rather unconventional.

In order to find out whether the unusual magnetic field dependence is also seen in the transport experiments, we have analyzed the *R* vs. *T* data of the thermal limit point contact obtained at different magnetic fields. The field-dependent *R*–*T* curves are shown in Fig. 6a. We have tracked the shift in transition at higher temperature with magnetic field to construct the *H*–*T* phase diagram. For the *R*–*T* curves at all magnetic fields it is seen that the transitions are rather broad. This limits us from having a precise measurement of the critical temperature ($T_c$). In order to be consistent, we have defined the $T_c$ as the temperature where the slopes of the *R*–*T* curve near the transition from the lower temperature side and that from the higher temperature side meet each other. This scheme is shown in the *inset* of Fig. 4f for zero magnetic fields while for higher magnetic fields (7–15 kG) is shown in Fig. 7 in the “Supplementary material”. We have applied the same scheme for the measurement of $T_c$ at all fields. As shown in Fig. 6b, the experimentally obtained *H*–*T* data shows dramatic deviation from the conventional *H*–*T* curve that is usually seen in conventional superconductors. These observations indicate the possibility of an unconventional component in the superconducting order parameter of the high-pressure phase^26^.

It should be noted that despite multiple attempts, a ballistic point contact could not be realized in this phase as during our efforts to reduce the contact diameter through controlled withdrawal of the tip, the effective pressure also decreased thereby causing a sudden disappearance of the pressure-induced phase. In order to show the reproducibility, we have presented a number of intermediate regime and thermal regime spectra in Figs. 3 and 4 in “Supplemental material” obtained at different points on the crystal.

## Conclusion

In conclusion, we have realized the pressure-enhanced superconducting phase of Sr-intercalated Bi$_2$Se$_3$ under hard point contacts and investigated both the low-pressure and the high-pressure phases spectroscopically. We found that while the low-pressure superconducting phase behaves like a conventional superconductor, the high-pressure phase has unusual magnetic properties. The critical current of the thermal point contacts formed with the high-pressure superconducting phase shows unusual rigidity with increasing magnetic field. Furthermore, The *H*–*T* phase diagram of the high-pressure phase shows dramatic deviation from a conventional convex shape. Such observations indicate the possibility of unconventional superconductivity (and, topological superconductivity) in the high-pressure superconducting phase of Sr-intercalated Bi$_2$Se$_3$. This work also demonstrates an unique way of spectroscopically probing pressure-induced or pressure-enhanced superconducting phases in new generation quantum materials.

## Supplementary information

Supplementary Information.
