# Supplementary material for: The pressure-enhanced superconducting phase of Srx–Bi2Se3 probed by hard point contact spectroscopy
Source: Sci Rep. 2021 Feb 18;11:4090. doi: 10.1038/s41598-021-83411-w (PMC7893176; doi:10.1038/s41598-021-83411-w)
Supplement: Supplementary file 1 — Supplementary Information. [file 41598_2021_83411_MOESM1_ESM.pdf]

Supplementary Material for the pressure-enhanced superconducting phase of Sr_x-Bi_2Se_3 probed by hard point contact spectroscopy

Ritesh Kumar[1], Aastha Vasdev[1], Shekhar Das[1], Sandeep Howlader[1], Karn S. Jat[2], Prakriti Neha[2], Satyabrata Patnaik[2], and Goutam Sheet[1*]

[1]*Department of Physical Sciences, Indian Institute of Science Education and Research Mohali, Mohali, Punjab, India and*

[2]*School of Physical Sciences, Jawaharlal Nehru University, New Delhi, India*

[*] goutam@iisermohali.ac.in

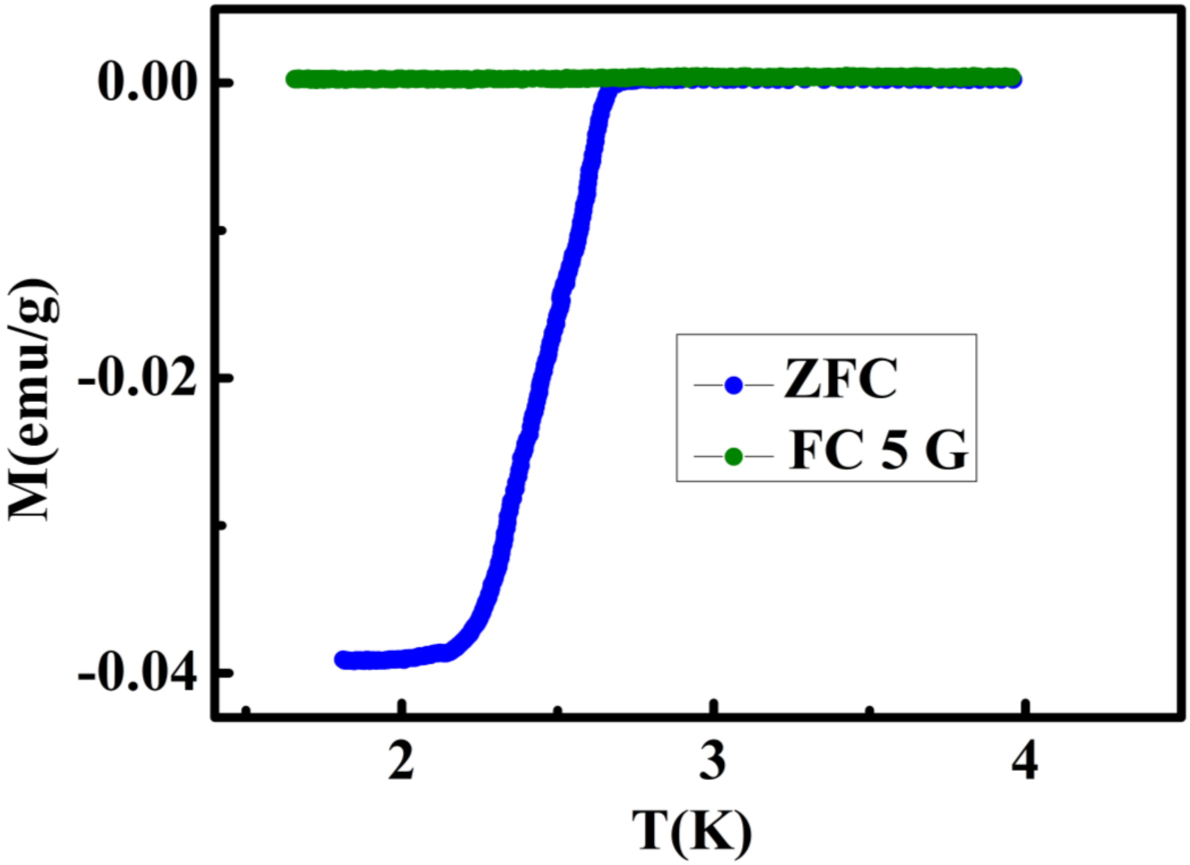

FIG. 1: Magnetization vs temperature (Zero field cooled (ZFC) and Field cooled (FC) data).

In Figure 1, the excitation field is applied parallel to the layers of sample. One can clearly see that in the parallel field configuration the transition width is much smaller (~ 0.5 K).

Figure 2 shows normalized pinning force density (F_p/F_{max}) as a function of reduced magnetic field (h/h_{irr}). In our sample, the calculated demagnetization factor is ≈ 0.23.

Here the Kramer's fitting for the formula

$F_p(h) = Ah^b(1-h)^c$

Provides the pinning coefficients as $A = 2.60174$, $b = 0.73433$, $c = 0.7011$

The peak position indicates the volume pinning tendency in the sample.

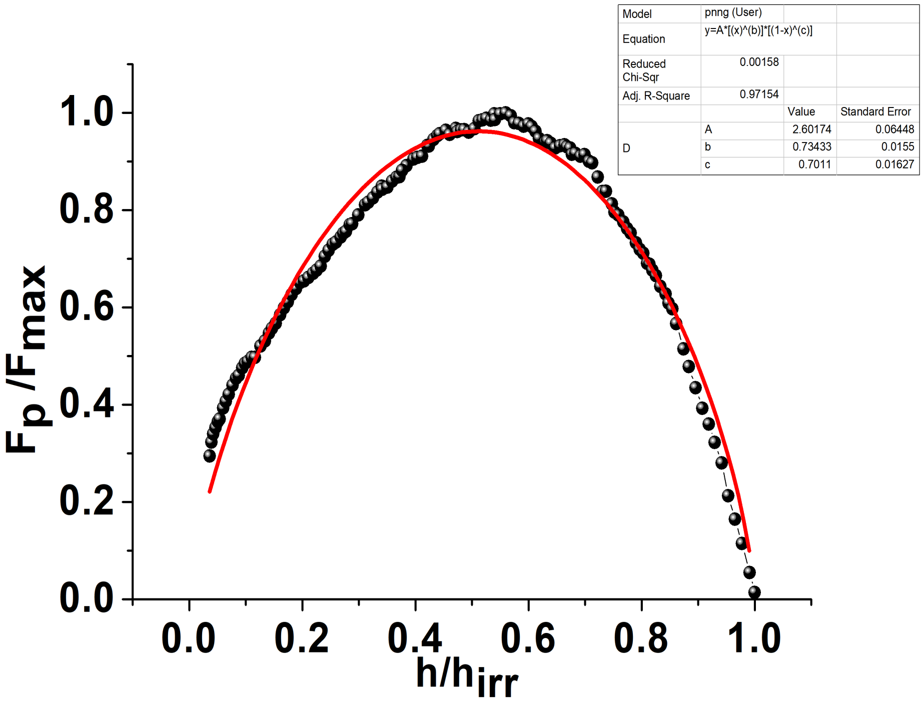

FIG. 2: Normalized pinning force density (F_p/F_{max}) as a function of reduced magnetic field (h/h_{irr}). Red solid line shows the Kramer's pinning model fitting.

Additional PCS data:

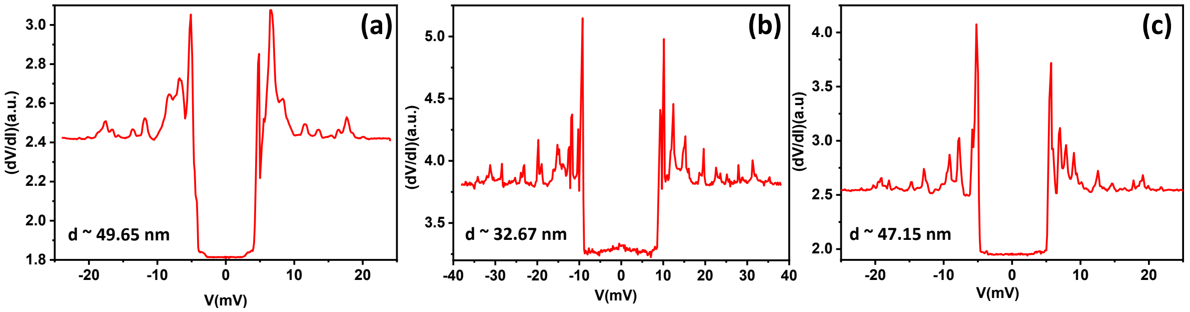

FIG. 3: (dV/dI) spectra obtained at different points of the point contact in the thermal regime. Where d is the contact diameter.

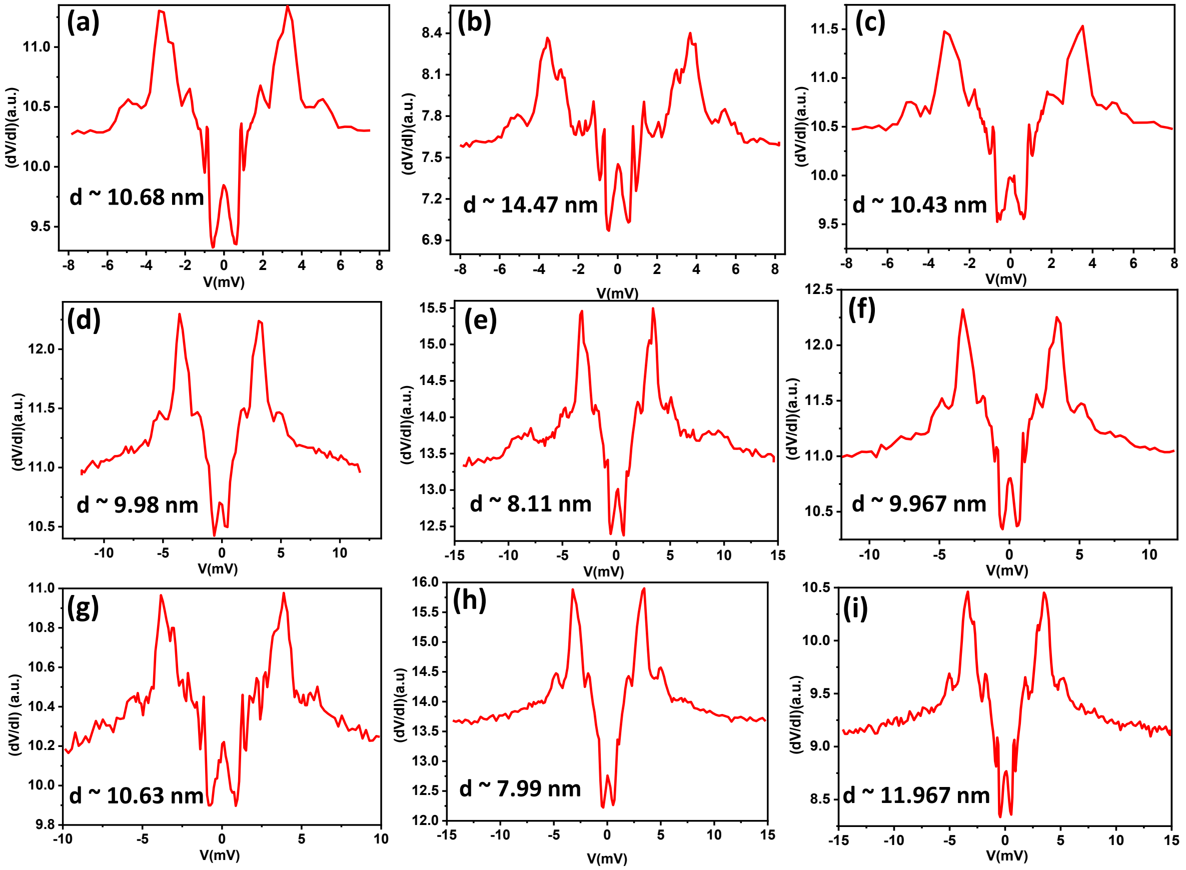

FIG. 4: (dV/dI) spectra obtained at different points of the point contact in the intermediate regime.

Scanning tunneling spectroscopy (STS) data: Fig.5 shows STS data obtained for $Sr_xBi_2Se_3$ at 370 mK along with the theoretical fit using Dyne's equation where the estimated superconducting gap is 0.31 meV.

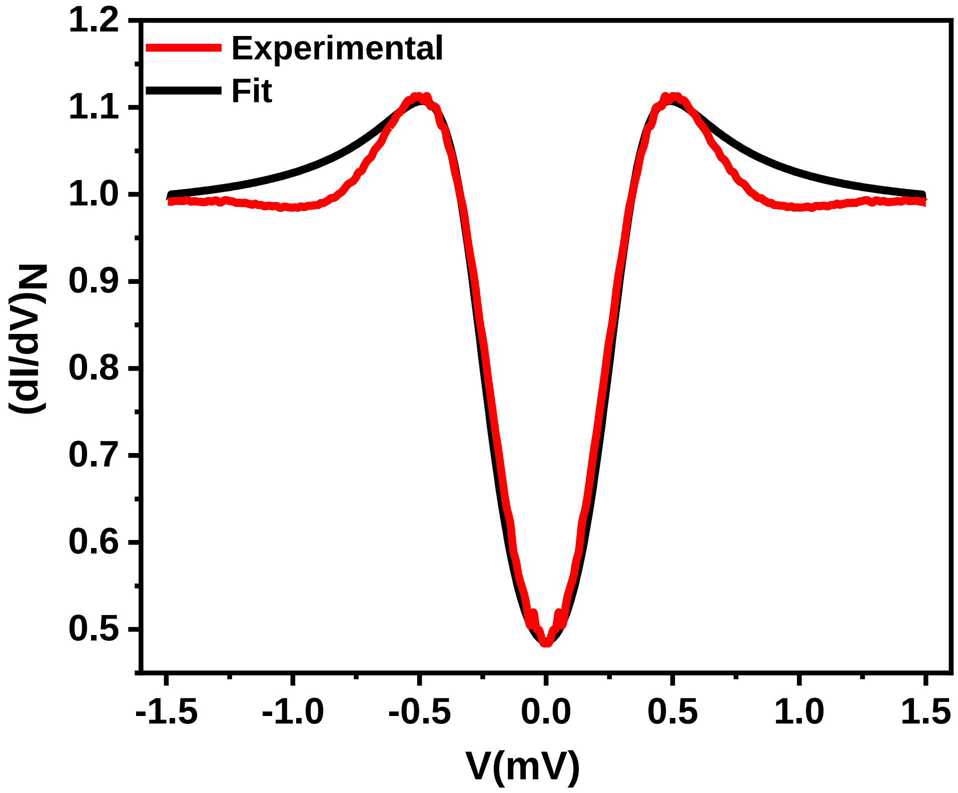

FIG. 5: Normalized scanning tunneling spectroscopy (STS) data with best theoretical fit using Dyne's equation.

Numerical simulation of a point contact in the intermediate regime: We have performed a numerical simulation to show how upturn arises in the RT curves shown in figure 2(f) measured for point contacts at zero dc bias. We first built a model to reproduce the features as shown figure 2 (a) in the manuscript where the inelastic contributions have been added to the BTK current. We have varied the temperature from 0.5 K to 37 K in the simulation and calculated the respective dV/dI vs. V spectrum as shown in figure 6(a). Form figure 6(a), we have extracted the zero bias resistance value for all the temperature and plot it in the respective figure 6 (b) which shows clear upturn in the R-T curve. Therefore, the upturn is not due to an artifact but due to intrinsic character of superconducting point contacts.

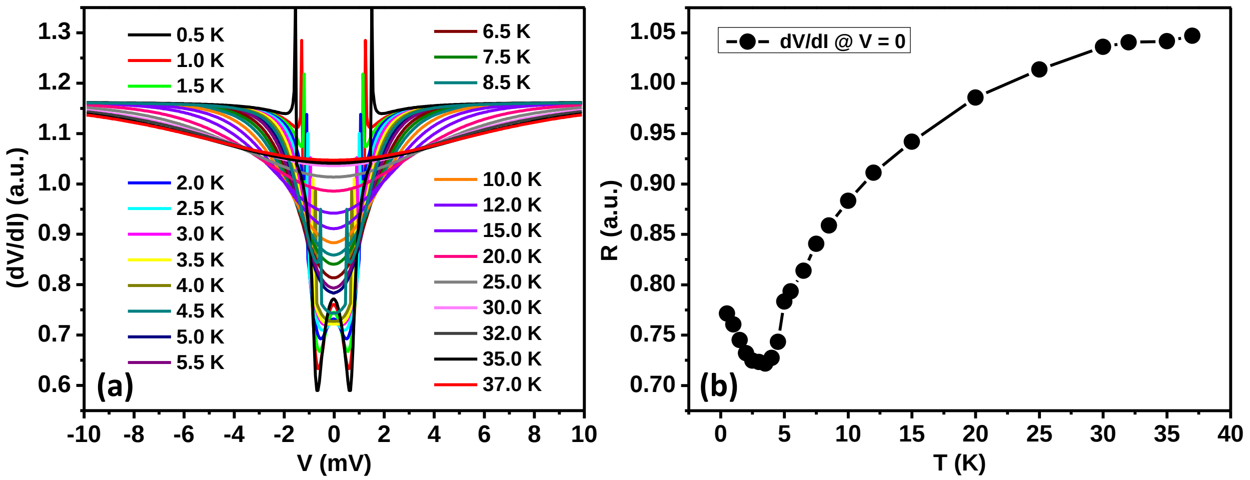

FIG. 6: (a) Temperature dependence of the calculated dV/dI vs. V spectrum. (b) Zero bias resistance (R) vs. Temperature (T) curve extracted from Fig. (a) .

Determination of T_c: Figure shows the measurement of the critical temperature (T_c) for the $R-T$ curves at magnetic fields 7 kG - 15 kG. We have defined the T_c as the temperature where the slopes of the $R-T$ curve near the transition from the lower temperature side and that from the higher temperature side meet each other.

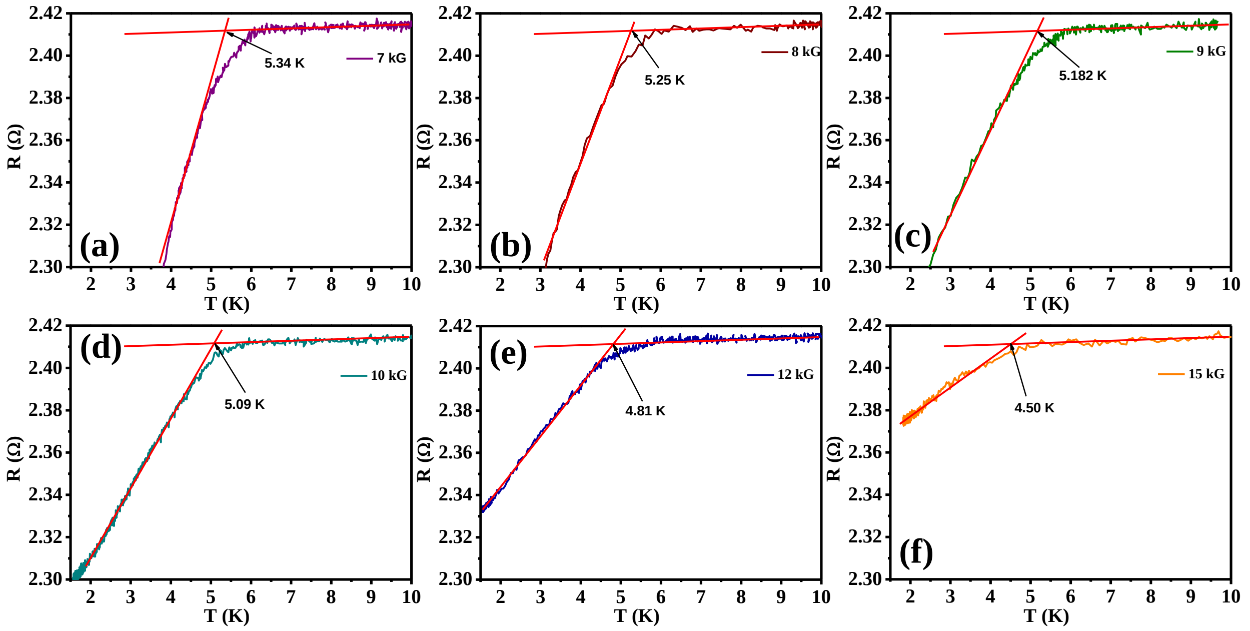

FIG. 7: (a-f)$R-T$ curves show the measurement of T_c for the magnetic fields 7 kG - 15 kG respectively.

TABLE I: **Fitting parameters used to fit spectra presented in Figure 3(a) in the main manuscript.**

**T (K)**	$\Delta(meV)$	**Z**	$\Gamma(meV)$
0.366	0.54	0.544	0.378
0.493	0.538	0.5275	0.376
0.636	0.526	0.0.523	0.374
0.792	0.513	0.499	0.374
0.929	0.51	0.485	0.368
1.1	0.481	0.4578	0.368
1.25	0.477	0.443	0.368
1.43	0.442	0.4	0.366
1.65	0.37	0.2995	0.36
1.95	0.3452	0.24	0.352
2.27	0.16	0.182	0.15
